# Supplementary material for: Machine Learning Based Assessment of Inguinal Lymph Node Metastasis in Patients with Squamous Cell Carcinoma of the Vulva
Source: J Clin Med. 2025 May 17;14(10):3510. doi: 10.3390/jcm14103510 (PMC12111910; doi:10.3390/jcm14103510)
Supplement: Supplementary file 1 [file jcm-14-03510-s001.zip › jcm-3529474-supplementary.pdf]

Supplementary Material

Title: Machine Learning Based Assessment of Inguinal Lymph Node Metastasis in Patients with Squamous Cell Carcinoma of the Vulva

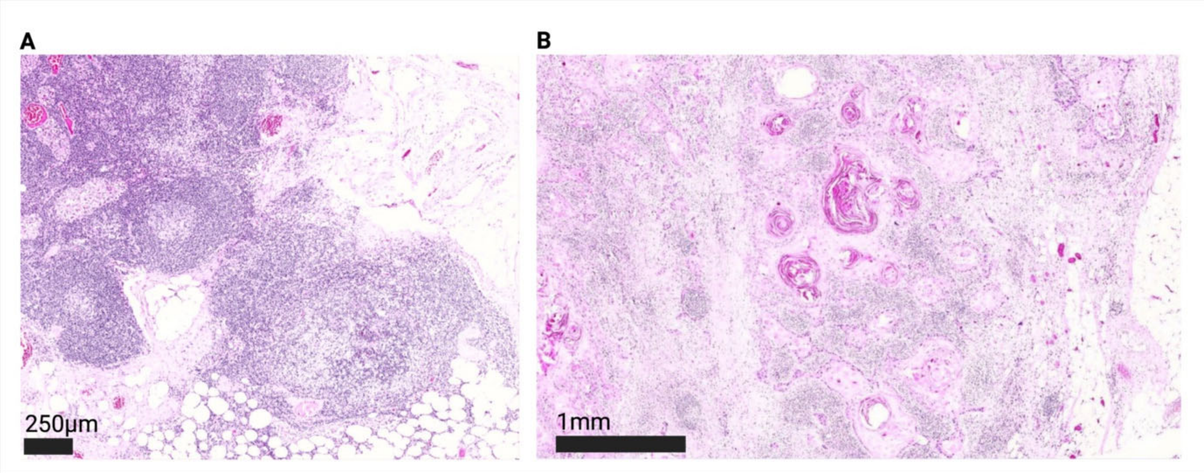

Figure S1. During the classifier training process, we defined positive/negative groin lymph node involvement as response variable. **A**: Regular lymph node architecture without metastatic lesions (negative lymph node involvement). **B**: Positive lymph node involvement showing infiltrates of keratinizing epithelial tumor cell clusters between architecturally disrupted lymphoid tissue. A-B: Hematoxylin and eosin staining.

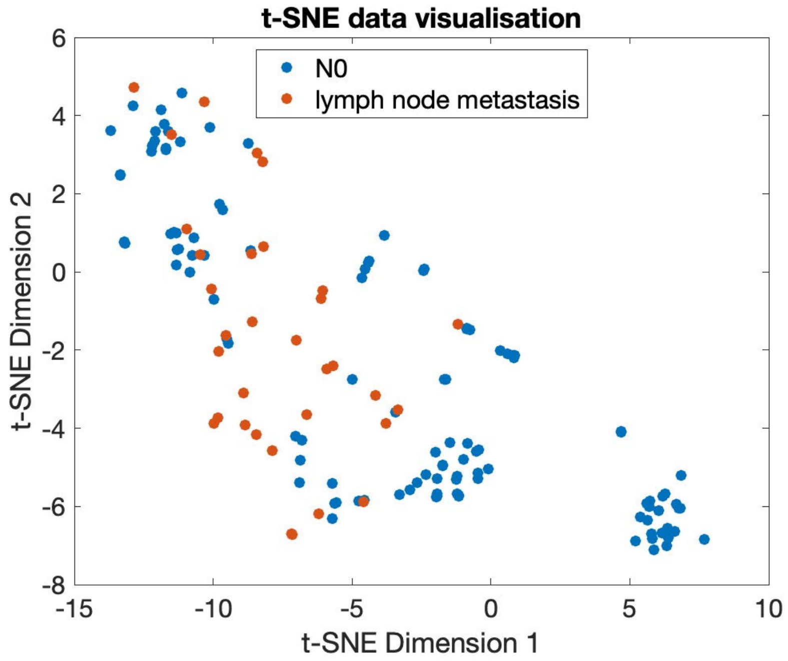

Figure S2. A t-SNE dimension allows initial data visualization. Blue dots = lymph node negative tumors; red dots = lymph node positive tumors.

Table S1. Listing of the extend of lymph node involvement within our study cohort.

extend of lymph node metastasis in our study cohort	total number of lymph node metastases n=33
Nmi/N1a	11 (33.4%)
N1b	5 (15.1%)
N2a	-
N2b	4 (12.1%)
N2c	13 (39.4%)

Table S2. Distribution of HPV-association (viral/non-viral tumorigenesis) as well as listing of different grades of histological grading within our cohort.

variables of interest	N=157
Histology: HPV-associated	25 (15.9%)
Histology: HPV-independent	54 (34.4%)
Histology: not otherwise specified (nos)	78 (49.7%)
Histological grade: I	75 (47.8%)
Histological grade: II	24 (15.3%)
Histological grade: III	33 (21.0%)
Histological grade: IV	25 (15.9%)

Table S3. Spearman correlation analysis of clinicopathological parameters with groin lymph node involvement.

**Spearman correlation:** Groin lymph node involvement in association to -	**r**	95% confidence interval
age	0,09540	-0.06682 to 0.2527
T stage	0,3211	0.1686 to 0.4585
infiltration depth (cm)	0,5066	0.3762 to 0.6174
tumor grading	0,2215	0.06259 to 0.3695

Table S4. Ranking of predictors and their corresponding Chi^2 values based on their feature importance (chi-square testing).

**feature rank**	**predictor variable**	**Chi^2-value**
**1**	lympho-vascular invasion	20.1908
**2**	infiltration depth	9.4645
**3**	perineural infiltration	7.9419
**4**	vascular infiltration	7.0037
**5**	T stage	5.7570
**6**	positive resection margin	5.4338
**7**	tumor grading	3.3769

Table S5. Metrices and classifier performance of our decision tree algorithm based on the external classifier validation process.

**key performance indicators of our tree classifier performance (external validation)**	overall accuracy = 83.9%
**no lymph node affection (N0):**	
TPR (true positive rate)	96.0%
FNR (false negative rate)	4.0%
PPV (positive predictive value)	85.7%
FDR (false discovery rate)	14.3%
**positive groin lymph node affection:**	
TPR (true positive rate)	33.3%
FNR (false negative rate)	66.7%
PPV (positive predictive value)	66.7%
FDR (false discovery rate)	33.3%
